# *n*-Butyl 2-(3-chloro-1,2-dihydro­pyrazin-2-yl­idene)-2-cyano­acetate

**DOI:** 10.1107/S1600536809031870

**Published:** 2009-08-15

**Authors:** Anita Stefańska, Tadeusz Ossowski, Łukasz Ponikiewski

**Affiliations:** a Faculty of Chemistry, University of Gdańsk, Sobieskiego 18/19, Gdańsk PL 80952, Poland; b Faculty of Chemistry, Gdańsk University Of Techology, Narutowicza 11/12, Gdańsk PL 80233, Poland

## Abstract

The title compound, C_11_H_12_ClN_3_O_2_, is essentially planar except for the *n*-but­oxy group [r.m.s. deviation from the least-squares plane = 0.0131 (1) Å for 11 non-H atoms]. An intra­molecular N—H⋯O inter­action results in the formation of an *S*(6) ring. The *n*-butoxy chain in the molecule is disordered over two sets of sites of equal occupancy.

## Related literature

For applications of this class of compounds, see: Matter *et al.* (2005); Kaliszan *et al.* (1985 ▶); Petrusewicz *et al.* (1992 ▶, 1993 ▶, 1995 ▶). For pyrazin­yl–pyrazyl­idene tautomerism, see: Pilarski *et al.* (1984 ▶). For related structures, see: Vishweshwar *et al.* (2000 ▶); Wardell *et al.* (2006 ▶). For the synthesis, see: Pilarski & Foks (1981 ▶, 1982 ▶).
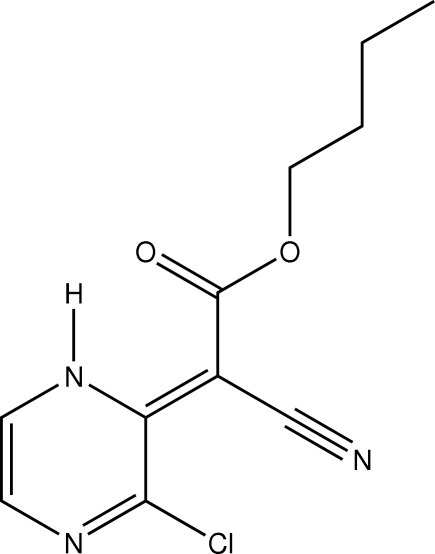


## Experimental

### 

#### Crystal data

C_11_H_12_ClN_3_O_2_
*M* *_r_* = 253.69
Monoclinic, 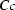

*a* = 4.918 (3) Å
*b* = 25.642 (7) Å
*c* = 10.573 (4) Å
β = 95.80 (3)°
*V* = 1326.7 (9) Å^3^
*Z* = 4
Mo *K*α radiation
μ = 0.28 mm^−1^
*T* = 120 K
0.57 × 0.11 × 0.07 mm

#### Data collection

Oxford Diffraction KM-4/Xcalibur diffractometer with a Sapphire2 (large Be window) detector
Absorption correction: Gaussian (*CrysAlis Pro*; Oxford Diffraction, 2007 ▶) *T*_min_ = 0.923, *T*_max_ = 0.987
4629 measured reflections
1687 independent reflections
1219 reflections with *I* > 2σ(*I*)
*R*_int_ = 0.034

#### Refinement

*R*[*F* ^2^ > 2σ(*F* ^2^)] = 0.047
*wR*(*F* ^2^) = 0.156
*S* = 1.04
1687 reflections
154 parameters
1 restraint
H atoms treated by a mixture of independent and constrained refinement
Δρ_max_ = 0.24 e Å^−3^
Δρ_min_ = −0.23 e Å^−3^
Absolute structure: Flack (1983 ▶), 466 Friedel pairs
Flack parameter: 0.05 (18)

### 

Data collection: *CrysAlis CCD* (Oxford Diffraction, 2006 ▶); cell refinement: *CrysAlis RED* (Oxford Diffraction, 2006 ▶); data reduction: *CrysAlis RED* (Oxford Diffraction, 2006 ▶); program(s) used to solve structure: *SHELXS97* (Sheldrick, 2008 ▶); program(s) used to refine structure: *SHELXL97* (Sheldrick, 2008 ▶); molecular graphics: *ORTEP-3 for Windows* (Farrugia, 1997 ▶); software used to prepare material for publication: *WinGX* (Farrugia, 1999 ▶).

## Supplementary Material

Crystal structure: contains datablocks global, I. DOI: 10.1107/S1600536809031870/ng2612sup1.cif

Structure factors: contains datablocks I. DOI: 10.1107/S1600536809031870/ng2612Isup2.hkl

Additional supplementary materials:  crystallographic information; 3D view; checkCIF report

## Figures and Tables

**Table 1:** Hydrogen-bond geometry (Å, °)

*D*—H⋯*A*	*D*—H	H⋯*A*	*D*⋯*A*	*D*—H⋯*A*
N2—H2*B*⋯O1	0.87 (2)	1.96 (5)	2.632 (6)	133 (6)

**Fig. 1.**
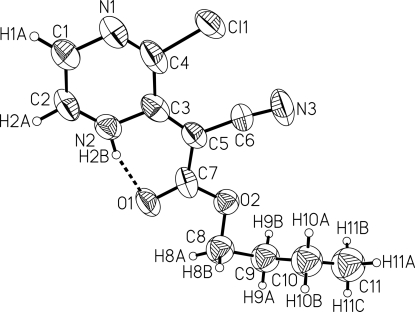
The moleculare structure of the title compound showing the atomic numbering scheme. Displacement ellipsoids are drawn at the 50% probability level.

## supplementary crystallographic information

### Comment

Heterocyclic compounds with active methylene moiety are known to have antiinflamatory (Petrusewicz *et al.*, 1995), analgesic (Kaliszan *et al.*, 1985) and large number of other pharmacological activities (Petrusewicz *et al.*, 1992). Numerous scientific publications confirm that pyrazine derivatives, obtained by nucleophilic substitution of chlorine atom in the pyrazine ring system with active methylene compound, posses above mentioned activity. Some of pyrazine C—H and N—H acids also demonstrated antithrombotic and antiplatelet activity (Petrusewicz *et al.*, 1993).

Such pharmacological activity (in group CH– andNH– acids) is possibly the result of acid character of particle and their structure, as in case of well known inhibitors of cyclooxygenase (Petrusewicz *et al.*, 1993). Structural analysis of pyrazine-acetonitrile derivatives shows pyrazinyl-pyrazylidene tautomerism (Pilarski *et al.*, 1984) but crystal of 2-butyl 2-(3-chloropyrazin- (1*H*)-ylidene)-2-cyanoacetateappears as NH-acid.

In the molecule of the title compound (Fig. 1) the bond lengths and angles characterizing the geometry of the pyrazines skeleton are typical for this group compounds (Vishweshwar *et al.* 2000; Wardell *et al.* 2006). The compound is essentially planar except for the n-butoxy group (r.m.s. deviation from the least-squares plane - 0.0131 (1) Å for 11 non-H atoms. An intramolecular N2—H2B···O1 contact generates a S(6) ring motif which stabilizes the molecular conformation. The n-butoxy chain in the molecule is disordered over two sets of sites in a 0.50 (1):0.50 (1) ratio.

### Experimental

2-butyl 2-(3-chloropyrazin-2(1*H*)-ylidene)-2-cyanoacetate was obtained bygeneral method described in papers (Pilarski *et al.*, 1981; Pilarski *et al.*, 1982). Crystallization of this compound from methanol forms a crystal.

### Refinement

Atoms O2, C8 (H8A, H8B), C9 (H9A, H9B), C10 (H10A, H10B), C11 (H11A, H11B, H11C) were disordered over two positions. During the refinement process the disordered atoms were refined with occupancies of 0.50 and 0.50. H atoms bonded to C were included in calculated positions and refined as riding on their parent C atom with C—H = 0.95 Å *U*_iso_(H) = 1.2 *U*_eq_(C) for aromatic, C—H = 0.99 Å *U*_iso_(H) = 1.2 *U*_eq_(C) for methylene and C—H = 0.98 Å *U*_iso_(H) = 1.5 *U*_eq_(C) for methyl H atoms. The H2B atom was located from difference Fourier map and refined isotropically resulting in N—H abond length 0.87 (2) Å *U*_iso_(H) = 0.10 (2) Å^2^. The carbon atoms C8, C9, C10, C11 and C8A, C9A, C10A, C11A were located from a difference map, fixed at 1.50 for C—C distance and refined with the *DFIX* restraint. The Flack (1983) parameter was refined explicity, with both TWIN and BASF parameters.

### Crystal data

**Table:**

C_11_H_12_ClN_3_O_2_	*F*(000) = 528
*M**_r_* = 253.69	*D*_x_ = 1.27 Mg m^−^^3^
Monoclinic, *C**c*	Mo *K*α radiation, λ = 0.71073 Å
Hall symbol: C -2yc	Cell parameters from 1500 reflections
*a* = 4.918 (3) Å	θ = 2.5–32.3°
*b* = 25.642 (7) Å	µ = 0.28 mm^−^^1^
*c* = 10.573 (4) Å	*T* = 120 K
β = 95.80 (3)°	Block, yellow
*V* = 1326.7 (9) Å^3^	0.57 × 0.11 × 0.07 mm
*Z* = 4	

### Data collection

**Table:**

Oxford Diffraction KM-4/Xcalibur diffractometer with a Sapphire2 (large Be window) detector	1687 independent reflections
graphite	1219 reflections with *I* > 2σ(*I*)
Detector resolution: 8.1883 pixels mm^-1^	*R*_int_ = 0.034
0.75° wide ω scans	θ_max_ = 25.5°, θ_min_ = 2.5°
Absorption correction: gaussian (*CrysAlis PRO*; Oxford Diffraction, 2007)	*h* = −3→5
*T*_min_ = 0.923, *T*_max_ = 0.987	*k* = −31→31
4629 measured reflections	*l* = −12→12

### Refinement

**Table:**

Refinement on *F*^2^	Secondary atom site location: difference Fourier map
Least-squares matrix: full	Hydrogen site location: inferred from neighbouring sites
*R*[*F*^2^ > 2σ(*F*^2^)] = 0.047	H atoms treated by a mixture of independent and constrained refinement
*wR*(*F*^2^) = 0.156	*w* = 1/[σ^2^(*F*_o_^2^) + (0.1833*P*)^2^] where *P* = (*F*_o_^2^ + 2*F*_c_^2^)/3
*S* = 1.04	(Δ/σ)_max_ < 0.001
1687 reflections	Δρ_max_ = 0.24 e Å^−^^3^
154 parameters	Δρ_min_ = −0.23 e Å^−^^3^
1 restraint	Absolute structure: Flack (1983), 466 Friedel pairs
Primary atom site location: structure-invariant direct methods	Flack parameter: 0.05 (18)

### Special details

**Table:**

Geometry. All e.s.d.'s (except the e.s.d. in the dihedral angle between two l.s. planes) are estimated using the full covariance matrix. The cell e.s.d.'s are taken into account individually in the estimation of e.s.d.'s in distances, angles and torsion angles; correlations between e.s.d.'s in cell parameters are only used when they are defined by crystal symmetry. An approximate (isotropic) treatment of cell e.s.d.'s is used for estimating e.s.d.'s involving l.s. planes.
Refinement. Refinement of *F*^2^ against ALL reflections. The weighted *R*-factor *wR* and goodness of fit *S* are based on *F*^2^, conventional *R*-factors *R* are based on *F*, with *F* set to zero for negative *F*^2^. The threshold expression of *F*^2^ > σ(*F*^2^) is used only for calculating *R*-factors(gt) *etc*. and is not relevant to the choice of reflections for refinement. *R*-factors based on *F*^2^ are statistically about twice as large as those based on *F*, and *R*- factors based on ALL data will be even larger.

### Fractional atomic coordinates and isotropic or equivalent isotropic displacement parameters (Å^2^)

**Table:**

	*x*	*y*	*z*	*U*_iso_*/*U*_eq_	Occ. (<1)
Cl1	0.7083 (3)	0.42260 (5)	0.30244 (15)	0.0753 (4)	
O1	0.0928 (8)	0.54438 (16)	0.6099 (3)	0.0731 (12)	
N1	0.4291 (11)	0.36488 (18)	0.4525 (5)	0.0827 (13)	
N2	0.1819 (9)	0.4445 (2)	0.5738 (4)	0.0612 (12)	
N3	0.7225 (16)	0.55178 (17)	0.2917 (6)	0.0874 (12)	
C1	0.2496 (17)	0.3549 (3)	0.5468 (7)	0.0905 (19)	
H1A	0.2102	0.3199	0.5681	0.109*	
C2	0.1303 (11)	0.3956 (3)	0.6086 (5)	0.0695 (16)	
H2A	0.0153	0.3889	0.6737	0.083*	
C3	0.3572 (10)	0.4596 (2)	0.4816 (4)	0.0548 (12)	
C4	0.4796 (11)	0.4144 (2)	0.4225 (4)	0.0649 (13)	
C5	0.4002 (10)	0.5136 (2)	0.4565 (4)	0.0569 (10)	
C6	0.5828 (11)	0.5330 (2)	0.3632 (5)	0.0617 (11)	
C7	0.2549 (12)	0.5528 (2)	0.5259 (5)	0.0626 (15)	
O2	0.3471 (16)	0.6051 (3)	0.5012 (7)	0.050 (2)*	0.5
C8	0.202 (2)	0.6474 (4)	0.5635 (11)	0.056 (3)*	0.5
H8A	0.2504	0.647	0.6566	0.067*	0.5
H8B	0.0014	0.6434	0.5458	0.067*	0.5
C9	0.296 (2)	0.6977 (4)	0.5064 (9)	0.070 (3)*	0.5
H9A	0.4939	0.7017	0.5331	0.084*	0.5
H9B	0.2014	0.7269	0.5444	0.084*	0.5
C10	0.253 (3)	0.7037 (4)	0.3607 (9)	0.072 (3)*	0.5
H10A	0.3655	0.6769	0.3231	0.086*	0.5
H10B	0.0594	0.6955	0.3331	0.086*	0.5
C11	0.316 (3)	0.7546 (5)	0.3052 (16)	0.095 (4)*	0.5
H11A	0.1591	0.7664	0.248	0.143*	0.5
H11B	0.4758	0.751	0.2573	0.143*	0.5
H11C	0.3558	0.7802	0.3734	0.143*	0.5
O2A	0.268 (2)	0.6013 (4)	0.4705 (9)	0.071 (3)*	0.5
C8A	0.111 (3)	0.6434 (5)	0.5272 (13)	0.080 (4)*	0.5
H8AA	0.1828	0.6493	0.6169	0.095*	0.5
H8AB	−0.0843	0.6336	0.5246	0.095*	0.5
C9A	0.142 (3)	0.6924 (5)	0.4496 (14)	0.102 (4)*	0.5
H9A1	0.0102	0.6892	0.3728	0.122*	0.5
H9A2	0.0775	0.7217	0.4998	0.122*	0.5
C10A	0.402 (3)	0.7092 (5)	0.4057 (13)	0.092 (3)*	0.5
H10C	0.4668	0.6798	0.3559	0.11*	0.5
H10D	0.5335	0.7124	0.4827	0.11*	0.5
C11A	0.433 (4)	0.7571 (5)	0.3292 (13)	0.089 (4)*	0.5
H11D	0.6227	0.7691	0.3421	0.133*	0.5
H11E	0.3116	0.7843	0.3561	0.133*	0.5
H11F	0.3845	0.7493	0.239	0.133*	0.5
H2B	0.150 (14)	0.4691 (19)	0.627 (5)	0.10 (2)*	

### Atomic displacement parameters (Å^2^)

**Table:**

	*U*^11^	*U*^22^	*U*^33^	*U*^12^	*U*^13^	*U*^23^
Cl1	0.0730 (7)	0.0881 (7)	0.0692 (6)	0.0104 (9)	0.0279 (5)	−0.0026 (8)
O1	0.085 (3)	0.067 (2)	0.073 (2)	0.003 (2)	0.035 (2)	0.0011 (18)
N1	0.098 (3)	0.070 (3)	0.085 (3)	0.007 (2)	0.036 (2)	0.001 (2)
N2	0.063 (3)	0.067 (3)	0.055 (2)	0.006 (2)	0.013 (2)	−0.003 (2)
N3	0.087 (3)	0.098 (3)	0.082 (3)	−0.002 (3)	0.034 (2)	0.021 (3)
C1	0.119 (5)	0.063 (3)	0.097 (4)	0.004 (3)	0.049 (4)	−0.001 (3)
C2	0.075 (4)	0.071 (4)	0.067 (3)	−0.004 (3)	0.028 (3)	0.002 (3)
C3	0.049 (3)	0.073 (3)	0.042 (2)	0.003 (2)	0.0044 (19)	−0.007 (2)
C4	0.063 (3)	0.080 (4)	0.053 (2)	0.009 (2)	0.014 (2)	−0.002 (2)
C5	0.055 (2)	0.065 (3)	0.052 (2)	−0.002 (2)	0.0109 (18)	0.0014 (17)
C6	0.058 (2)	0.073 (3)	0.056 (2)	0.002 (2)	0.014 (2)	0.002 (2)
C7	0.069 (3)	0.060 (4)	0.062 (3)	−0.007 (3)	0.018 (3)	0.004 (3)

### Geometric parameters (Å, °)

**Table:**

Cl1—C4	1.791 (5)	C9—H9A	0.99
O1—C7	1.271 (7)	C9—H9B	0.99
N1—C4	1.338 (7)	C10—C11	1.478 (14)
N1—C1	1.421 (8)	C10—H10A	0.99
N2—C2	1.338 (9)	C10—H10B	0.99
N2—C3	1.419 (8)	C11—H11A	0.98
N2—H2B	0.87 (2)	C11—H11B	0.98
N3—C6	1.175 (8)	C11—H11C	0.98
C1—C2	1.392 (10)	O2A—C8A	1.489 (17)
C1—H1A	0.95	C8A—C9A	1.516 (14)
C2—H2A	0.95	C8A—H8AA	0.99
C3—C5	1.428 (7)	C8A—H8AB	0.99
C3—C4	1.475 (7)	C9A—C10A	1.469 (14)
C5—C7	1.472 (8)	C9A—H9A1	0.99
C5—C6	1.486 (7)	C9A—H9A2	0.99
C7—O2A	1.379 (11)	C10A—C11A	1.487 (14)
C7—O2	1.448 (10)	C10A—H10C	0.99
O2—C8	1.489 (13)	C10A—H10D	0.99
C8—C9	1.514 (12)	C11A—H11D	0.98
C8—H8A	0.99	C11A—H11E	0.98
C8—H8B	0.99	C11A—H11F	0.98
C9—C10	1.541 (12)		
			
C4—N1—C1	118.8 (5)	C10—C9—H9A	107.8
C2—N2—C3	126.2 (5)	C8—C9—H9B	107.8
C2—N2—H2B	117 (5)	C10—C9—H9B	107.8
C3—N2—H2B	114 (5)	H9A—C9—H9B	107.2
C2—C1—N1	121.0 (6)	C11—C10—C9	118.2 (10)
C2—C1—H1A	119.5	C11—C10—H10A	107.8
N1—C1—H1A	119.5	C9—C10—H10A	107.8
N2—C2—C1	118.3 (5)	C11—C10—H10B	107.8
N2—C2—H2A	120.9	C9—C10—H10B	107.8
C1—C2—H2A	120.9	H10A—C10—H10B	107.1
N2—C3—C5	120.3 (5)	C7—O2A—C8A	115.7 (8)
N2—C3—C4	112.3 (5)	O2A—C8A—C9A	107.3 (11)
C5—C3—C4	127.5 (5)	O2A—C8A—H8AA	110.2
N1—C4—C3	123.5 (4)	C9A—C8A—H8AA	110.2
N1—C4—Cl1	115.1 (4)	O2A—C8A—H8AB	110.2
C3—C4—Cl1	121.4 (4)	C9A—C8A—H8AB	110.2
C3—C5—C7	118.7 (4)	H8AA—C8A—H8AB	108.5
C3—C5—C6	124.0 (4)	C10A—C9A—C8A	123.5 (13)
C7—C5—C6	117.3 (5)	C10A—C9A—H9A1	106.5
N3—C6—C5	175.5 (5)	C8A—C9A—H9A1	106.5
O1—C7—O2A	120.7 (6)	C10A—C9A—H9A2	106.5
O1—C7—O2	120.9 (5)	C8A—C9A—H9A2	106.5
O2A—C7—O2	19.8 (5)	H9A1—C9A—H9A2	106.5
O1—C7—C5	127.1 (5)	C9A—C10A—C11A	123.6 (13)
O2A—C7—C5	111.0 (6)	C9A—C10A—H10C	106.4
O2—C7—C5	111.5 (5)	C11A—C10A—H10C	106.4
C7—O2—C8	115.0 (7)	C9A—C10A—H10D	106.4
O2—C8—C9	105.3 (8)	C11A—C10A—H10D	106.4
O2—C8—H8A	110.7	H10C—C10A—H10D	106.5
C9—C8—H8A	110.7	C10A—C11A—H11D	109.5
O2—C8—H8B	110.7	C10A—C11A—H11E	109.5
C9—C8—H8B	110.7	H11D—C11A—H11E	109.5
H8A—C8—H8B	108.8	C10A—C11A—H11F	109.5
C8—C9—C10	117.8 (9)	H11D—C11A—H11F	109.5
C8—C9—H9A	107.8	H11E—C11A—H11F	109.5
			
C4—N1—C1—C2	−0.9 (10)	C6—C5—C7—O1	−179.2 (5)
C3—N2—C2—C1	−2.4 (9)	C3—C5—C7—O2A	−166.0 (6)
N1—C1—C2—N2	2.2 (11)	C6—C5—C7—O2A	13.6 (8)
C2—N2—C3—C5	−178.1 (5)	C3—C5—C7—O2	172.8 (5)
C2—N2—C3—C4	1.2 (7)	C6—C5—C7—O2	−7.6 (8)
C1—N1—C4—C3	−0.4 (8)	O1—C7—O2—C8	−10.3 (11)
C1—N1—C4—Cl1	−179.9 (5)	O2A—C7—O2—C8	85.0 (19)
N2—C3—C4—N1	0.3 (7)	C5—C7—O2—C8	177.5 (7)
C5—C3—C4—N1	179.5 (5)	C7—O2—C8—C9	−170.4 (8)
N2—C3—C4—Cl1	179.8 (3)	O2—C8—C9—C10	56.8 (12)
C5—C3—C4—Cl1	−1.0 (7)	C8—C9—C10—C11	173.4 (12)
N2—C3—C5—C7	−1.4 (6)	O1—C7—O2A—C8A	7.6 (13)
C4—C3—C5—C7	179.5 (5)	O2—C7—O2A—C8A	−89.0 (19)
N2—C3—C5—C6	179.1 (5)	C5—C7—O2A—C8A	175.8 (9)
C4—C3—C5—C6	−0.1 (8)	C7—O2A—C8A—C9A	−177.5 (10)
C3—C5—C6—N3	18E1(10)	O2A—C8A—C9A—C10A	−42.8 (19)
C7—C5—C6—N3	0(8)	C8A—C9A—C10A—C11A	179.8 (14)
C3—C5—C7—O1	1.2 (8)		

### Hydrogen-bond geometry (Å, °)

**Table:**

*D*—H···*A*	*D*—H	H···*A*	*D*···*A*	*D*—H···*A*
N2—H2B···O1	0.87 (2)	1.96 (5)	2.632 (6)	133 (6)
